# Clinical Features and Risk Factors for Active Tuberculosis in Takayasu Arteritis: A Single-Center Case-Control Study

**DOI:** 10.3389/fimmu.2021.749317

**Published:** 2021-10-29

**Authors:** Jiawei Zhou, Ruoyu Ji, Rui Zhu, Jingya Zhou, Jing Li, Xinping Tian, Yuexin Chen, Yuehong Zheng

**Affiliations:** ^1^ Department of Vascular Surgery, Peking Union Medical College Hospital, Chinese Academy of Medical Sciences and Peking Union Medical College, Beijing, China; ^2^ Peking Union Medical College Hospital, Chinese Academy of Medical Sciences and Peking Union Medical College, Beijing, China; ^3^ Department of Medical Record, Peking Union Medical College Hospital, Chinese Academy of Medical Sciences and Peking Union Medical College, Beijing, China; ^4^ Department of Rheumatology, Chinese Academy of Medical Sciences and Peking Union Medical College, Beijing, China

**Keywords:** Takayasu arteritis, tuberculosis, risk factor (RF), hsCRP, T-SPOT.TB

## Abstract

**Backgrounds:**

Takayasu arteritis (TAK) is a chronic, granulomatous vasculitis correlated with tuberculosis (TB). The two diseases share similar pathological characteristics and clinical manifestations which increase the difficulty to diagnose. Active tuberculosis (ATB) has implications for treatment strategies in TAK patients. Therefore, the investigation of clinical features and potential risk factors of ATB in TAK patients is vital.

**Methods:**

The study reviewed hospitalized patients diagnosed with TAK in our hospital from 2008, to 2021. TAK patients with ATB were enrolled as the case group. The control group was randomly selected in a 3:1 ratio. The clinical characteristics of TAK patients with and without ATB were compared. Multivariate logistic regression analysis was performed to determine risk factors for ATB in TAK patients.

**Results:**

We reviewed 1,789 patients and ultimately identified 30 (1.7%) ATB cases. TAK patients with ATB were more prone to develop symptoms including fever (p=0.001), fatigue (p=0.003), cough (p=0.037), expectoration (p<0.001), weight loss (p=0.003), and night sweating (p<0.001). Increased level of hypersensitive C reactive protein (hsCRP, p=0.001), decreased level of albumin (p=0.031), and higher positive rate of T-SPOT.TB test (p<0.001) were observed in the case group. Multivariate logistic regression analysis revealed that hsCRP >8 mg/L (OR 9.108; 95% CI, 1.096–75.711; p=0.041) and positive T-SPOT.TB result (OR 68.669; 95% CI, 7.291–646.738; p<0.001) were risk factors for ATB in TAK patients. The proportion of patients undergoing subsequent surgery for Takayasu arteritis was lower in patients with ATB (p<0.001).

**Conclusion:**

Our study suggested that the diagnosis of ATB should be considered when TAK patients experienced symptoms including fever, fatigue, weight loss, *etc.* hsCRP >8 mg/L and positive T-SPOT.TB result were identified as independent risk factors for ATB in TAK patients.

## 1 Introduction

Takayasu arteritis (TAK) is a chronic, granulomatous, large-vessel vasculitis involving the aorta and its major branches. TAK exhibits a predilection to occur in young women, comprising 75–97% of the reported cases (1). The prevalence of TAK varies by geographical location, which was estimated to be about 40 cases per million in Asia, while only 5 cases per million in the UK (2, 3).

It has been reported that TAK is correlated with tuberculosis (TB), which is also a considerable disease burden worldwide (4). Pathological characteristics of both diseases are granulomas and caseous necrosis, and their constitutional symptoms overlap (5). Most TAK patients require immunosuppressive treatment, which could lead to the dissemination of TB in patients with active tuberculosis (ATB). In turn, ATB may also contribute to the formation of saccular aneurysms or pseudoaneurysms, causing the deterioration of TAK patients (6). Moreover, TAK patients with major blood vessel involvement may require surgical intervention, while ATB is an absolute contraindication for surgery. Hence, the early and accurate recognition of ATB in TAK patients is essential for guidance on subsequent medical or surgical treatments, but information regarding this issue is still insufficient. Most of the studies were case reports or retrospective study with limited sample size (7–9). The studies with appreciable sample size focused on clinical characteristics in patients with TAK with and without TB infection, which did not distinguish ATB from other states of TB infection: latent tuberculosis infection (LTBI) or previous tuberculosis (PTB) (10, 11).

Moreover, to the best of our knowledge, there is no study assessing risk factors for ATB in TAK patients. Therefore, a case-control study was designed and conducted to investigate the clinical features and to explore risk factors for ATB in TAK patients, which could be helpful for the early recognition of patients at high risk for ATB, as well as timely intervention.

## 2 Methods

### 2.1 Participants

A retrospective review of the medical record of all hospitalized patients diagnosed with TAK in Peking Union Medical College Hospital (PUMCH) from January 1, 2008, to April 30, 2021, was performed. The diagnosis of TAK was made using the American College of Rheumatology 1990 criteria for the classification of Takayasu arteritis (12). For patients who were suspected to be infected with *M. tuberculosis* according to their clinical manifestations, full laboratory tests were performed including, but not limited to, serological tests (blood routine test, biochemical indicators, T-SPOT.TB test, etc.), laboratory etiological detections (blood and sputum culture, etc.), and histological examinations (intestinal mucosal biopsy, etc.). The diagnosis of ATB was comprehensively evaluated based on clinical symptoms (such as fever, fatigue, cough, night sweating, and weight loss), chest X-ray or computed tomography (CT), and laboratory examinations mentioned above with the help of consultation by the experienced experts of infectious diseases following the clinical practice guideline developed by the department of infectious diseases of Peking Union Medical College Hospital. The guideline was established and modified based on the “Diagnostic Standards and Classification of Tuberculosis in Adults and Children” published in 2000 by the Council of the Infectious Disease Society of America as shown in **Table S1** (13). The patients in the control group were extracted by random sampling technique from the remaining TAK patients without ATB, which was confirmed by a follow-up period starting at the first admission for TAK and ending at the date of the latest medical record.

### 2.2 Data Collection

**Figure 1:**
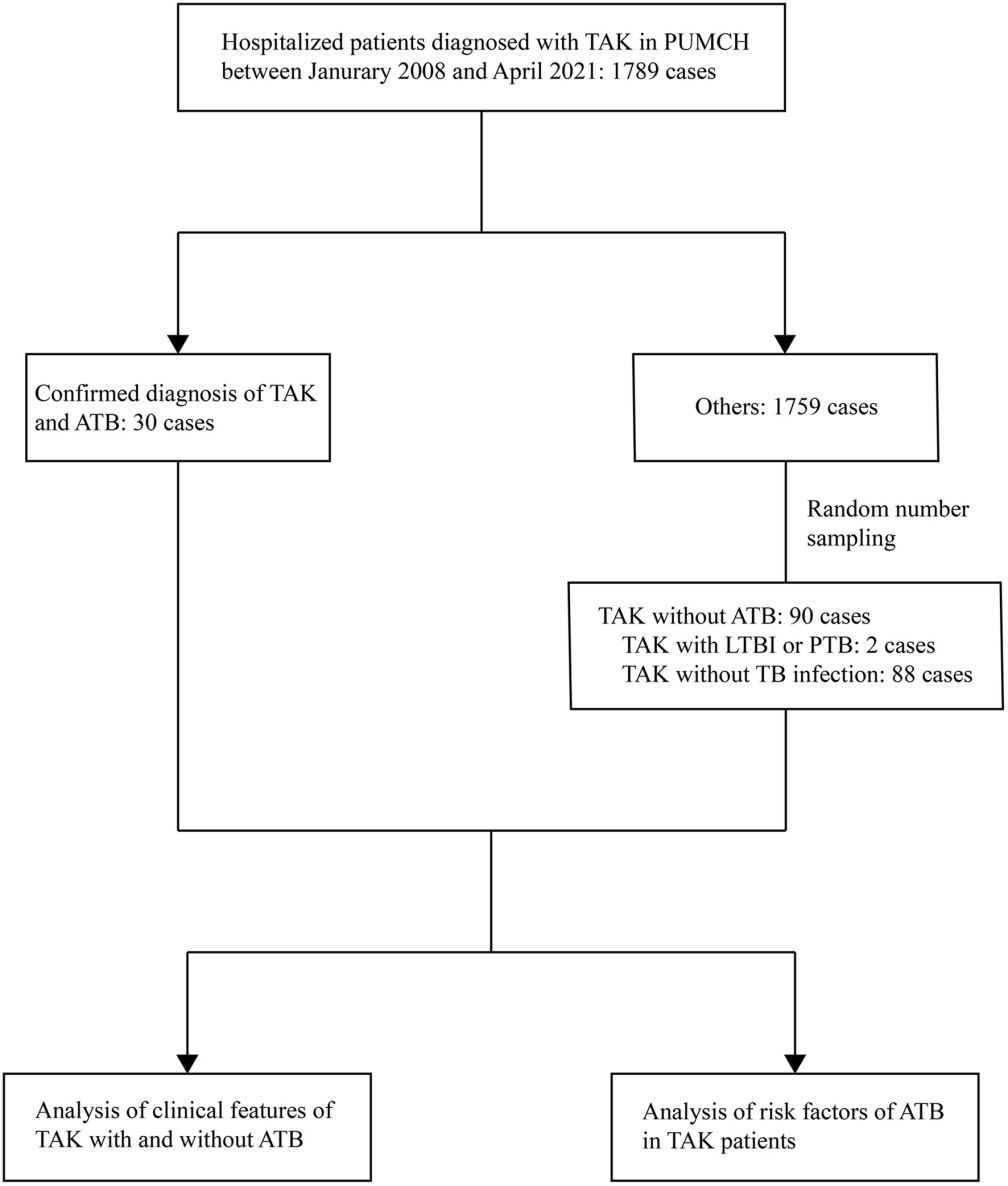
Flow chart of the study. TAK, Takayasu arteritis; PUMCH, Peking Union Medical College Hospital; ATB, active tuberculosis; LTBI, latent tuberculosis infection; PTB, previous tuberculosis.

The demographic features, past medical history, NUMANO classification of TAK, involved vessels, previous medications of TAK, clinical symptoms, and laboratory test results were collected and analyzed (14). Data regarding subsequent surgical interventions of TAK were collected during the follow-up period. The flowchart of the study is shown in **Figure 1**.

### 2.3 Ethic Review

This study complied with the Declaration of Helsinki and was approved by the Ethics Committee of PUMCH (ethics approval number: S-715). Informed consent was obtained from all patients enrolled.

### 2.4 Statistical Analyses

Continuous variables with normal distribution were described as mean ± standard deviation (SD), and those with non-normal distribution were presented as median and interquartile range (IQR) or median and range. Categorical variables were described as absolute value and percentages. Comparisons of continuous variables were performed with Student’s t-test or Mann–Whitney U-test, as appropriate. Categorical variables were compared by chi-square test or Fisher exact test, as appropriate. Basic demographic data and laboratory indices with P value < 0.10 between two groups were included in univariate logistic regression analysis. In the analysis, the lower or upper limit of the laboratory indices of our medical center was utilized to convert continuous variables into categorical variables with two groups. Variables with P value < 0.10 in univariate analysis were further included in multivariate analysis. All data analyses were performed using SPSS 25.0 for windows (SPSS institute, Chicago, IL, USA), and P value < 0.05 was considered statistically significant.

## 3 Results

### 3.1 General Data

Of a total of 120 TAK patients enrolled in this study, 93 (77.5%) patients were female. The median age of the patients was 27.0 years (range, 4.0 to 62.0 years) with a median disease duration of 10.5 (3.0, 36.0) months before admission. According to angiographic findings, 24 (20.0%), 10 (8.3%), 11 (9.2%), 5 (4.2%), 15 (12.5%), and 55 (45.8%) patients were classified as NUMANO type I, IIa, IIb, III, IV, and V, respectively. Thirty (25.0%) of included patients were confirmed with the diagnosis of ATB during the disease course of TAK, while the remaining 90 patients (75.0%) without ATB were categorized into the control group with a median follow-up period of 21.3 (5.8, 42.7) months. Detailed involved arteries are shown in **Table S2**.

### 3.2 Clinical Comparisons of TAK Patients With and Without ATB

#### 3.2.1 Patient Characteristics

Basic demographic data including age, sex, body mass index (BMI), and residential location did not differ significantly between two groups (**Table 1**). Patients with ATB had shorter disease course [6 (2, 12) *vs.* 12 (4, 36), p=0.013] compared with patients without ATB. In addition, there were no significant differences in comorbidities and the NUMANO classification between patients with and without ATB.

**Table 1:** Patient characteristics and management of TAK patients with and without ATB.

Variables	TAK with ATB (n = 30)	TAK without ATB (n = 90)	P value
**Demographic characteristics**			
Sex (male, %)	9 (30.0%)	18 (20.0%)	0.313
Age (years, median, range)	24.0 (4.0–62.0)	27.5 (9.0–52.0)	0.861
BMI (kg/m^2^, mean ± SD)	20.43 ± 4.28	21.06 ± 4.29	0.491
Residential location (Urban, %)	11 (36.7%)	39 (43.3%)	0.521
Disease course before admission (months, median, IQR)	6 (2, 12)	12 (4, 36)	0.013
**Comorbid diseases**			
Hypertension (%)	4 (13.3%)	4 (4.4%)	0.106
Diabetes mellitus (%)	1 (3.3%)	3 (3.3%)	1.000
Stroke (%)	0 (0.0%)	1 (1.1%)	1.000
Angina (%)	1 (3.3%)	1 (1.1%)	0.439
Renal dysfunction (%)	1 (3.3%)	1 (1.1%)	0.439
Heart failure (%)	0 (0.0%)	2 (2.2%)	1.000
Hepatitis B infection (%)	0 (0.0%)	2 (2.2%)	1.000
Hyperlipidemia (%)	1 (3.3%)	3 (3.3%)	1.000
**NUMANO classification**			
I (%)	4 (13.3%)	20 (22.2%)	0.290
IIa (%)	3 (10.0%)	7 (7.8%)	
IIb (%)	1 (3.3%)	10 (11.1%)	
III (%)	3 (10.0%)	2 (2.2%)	
IV (%)	3 (10.0%)	12 (13.3%)	
V (%)	16 (53.3%)	39 (43.3%)	
**Previous medications of TAK**			
Use of glucocorticoid (%)^a^	12 (13.3%)	50 (13.3%)	0.671
Maximal dosage (mg, median, IQR)	35 (20, 50)	30 (40, 50)	0.391
Duration (months, median, IQR)	1.5 (0.5, 7.8)	6.5 (2.0, 19.5)	0.017
Use of immunosuppressant (%)	5 (16.7%)	33 (36.7%)	0.041
**Subsequent surgical interventions of TAK**			
Surgery treatment (%)	5 (16.7%)	48 (53.5%)	<0.001
Dilatation (%)	1 (3.3%)	16 (17.8%)	0.068
Stent implantation (%)	2 (6.7%)	7 (7.8%)	1.000
Angiography (%)	2 (6.7%)	25 (27.8%)	0.016

a All forms of glucocorticoid were converted to the equivalent dosage of prednisone.

#### 3.2.2 Previous Medications of TAK

In terms of treatment of TAK (**Table 1**), 12 (40.0%) patients with ATB and 50 (55.6%) patients without ATB were previously treated with glucocorticoid. Patients with ATB underwent shorter duration of glucocorticoid treatment [1.5 (0.5, 7.8) *vs.* 6.5 (2.0, 19.5), p=0.017]. Additionally, patients without ATB were more likely to receive the immunosuppressant treatment (36.7 *vs.* 16.7%, p=0.041).

#### 3.2.3 Symptoms and Signs

TAK-related symptoms or signs did not exhibit significant difference between patients with and without ATB (**Table 2**). However, TAK patients with ATB were more prone to develop symptoms including fever (63.3 *vs.* 28.9%, p=0.001), fatigue (40.0 *vs.* 14.4%, p=0.003), cough (30.0 *vs.* 13.3%, p=0.037), expectoration (33.3 *vs.* 4.4%, p<0.001), weight loss (20.0 *vs.* 2.2%, p=0.003), and night sweating (60.0 *vs.* 16.7%, p<0.001).

**Table 2:** Clinical presentation and laboratory results of TAK patients with and without ATB.

Variables	TAK with ATB (n = 30)	TAK without ATB (n = 90)	P value
**Systemic symptoms**			
Fever (%)	19 (63.3%)	26 (28.9%)	0.001
Fatigue (%)	12 (40.0%)	13 (14.4%)	0.003
Cough (%)	9 (30.0%)	12 (13.3%)	0.037
Expectoration (%)	10 (33.3%)	4 (4.4%)	<0.001
Night sweating (%)	6 (20.0%)	2 (2.2%)	0.003
Weight loss (%)	18 (60.0%)	15 (16.7%)	<0.001
**Symptoms and signs related to TAK**			
Joint pain (%)	6 (20.0%)	9 (10.0%)	0.200
Dizziness (%)	10 (33.3%)	32 (35.6%)	0.825
Weak pulse (%)	15 (50.0%)	52 (57.8%)	0.458
BP discrepancies between arms (%)	14 (46.7%)	29 (32.2%)	0.153
Intermittent claudication (%)	1 (3.3%)	1 (1.1%)	0.439
**Laboratory tests**			
ESR (mm/h, median, IQR)	24 (16, 94)	16 (7, 52)	0.058
hsCRP (mg/L, median, IQR)	19.19 (5.97, 115.34)	3.19 (0.86, 25.13)	0.001
WBC (10^9^/L, median, IQR)	7.73 (6.37, 10.46)	7.64 (5.87, 10.25)	0.671
NEUT (10^9^/L, median, IQR)	5.18 (4.19, 6.69)	4.94 (3.45, 7.08)	0.654
LYMPH (10^9^/L, median, IQR)	1.98 (1.35, 2.59)	1.77 (1.35, 2.60)	0.557
PLT (10^9^/L, median, IQR)	312.5 (240.3, 383.0)	267.5 (214.8, 354.3)	0.193
HGB (g/L, median, IQR)	120.5 (107.5, 132.0)	118 (106.8, 138.3)	0.858
Alb (g/L, median, IQR)	38.5 (34.5, 42.3)	40.5 (38.0, 43.0)	0.031
AST (U/L, median, IQR)	17.0 (14.5, 24.0)	16.0 (14.0, 22.0)	0.525
ALT (U/L, median, IQR)	14.5 (11.0, 21.3)	14.0 (9.0, 20.0)	0.414
Tbil (μmol/L, median, IQR)	8.05 (6.13, 10.93)	9.10 (7.65, 12.68)	0.104
LDH (U/L, median, IQR)	174.0 (139.5, 202.0)	181.0 (145.5, 223.0)	0.569
Creatinine (μmol/L, median, IQR)	60.0 (48.8, 68.3)	59.0 (49.8, 69.0)	0.911
IgG (g/L, median, IQR)	11.75 (10.03, 13.67)	10.42 (8.49, 14.40)	0.228
IgA (g/L, median, IQR)	2.60 (1.71, 3.51)	2.40 (1.62, 3.36)	0.686
IgM (g/L, median, IQR)	1.17 (0.76, 1.64)	1.33 (1.00, 1.87)	0.171
Positive T-SPOT.TB (%)	15 (62.5%) (n=24)	3 (7.3%) (n=41)	<0.001
T-SPOT.TB value A (SFC/106PBMC, median, IQR)	48 (0, 220)	0 (0, 0)	<0.001
T-SPOT.TB value B (SFC/106PBMC, median, IQR)	76 (0, 200)	0 (0, 0)	<0.001

ESR, erythrocyte sedimentation rate; hsCRP, hypersensitive C reactive protein; WBC, white blood cell count; NEUT, neutrophil count; LYMPH, lymphocyte count; PLT, platelet count; HGB, hemoglobin; Alb, albumin; AST, aspartate aminotransferase; ALT, alanine aminotransferase; Tbil, total bilirubin; LDH, lactate dehydrogenase; IgG, immunoglobulin G; IgA, immunoglobulin A; IgM, immunoglobulin M; SFC, spot-forming cells; PBMC, peripheral blood mononuclear cells.

#### 3.2.4 Laboratory Tests

Compared with patients without ATB, ATB patients had increased level of hypersensitive C reactive protein [hsCRP, 19.19 (5.97, 115.34) *vs.* 3.19 (0.86, 25.13) mg/L, p=0.001], decreased level of albumin [38.5 (34.5, 42.3) *vs.* 40.5 (38.0, 43.0), p=0.031], and higher positive rate of T-SPOT.TB test (62.5 *vs.* 7.3%, p<0.001) (**Table 2**). The remaining laboratory results revealed no significant difference between the two groups.

### 3.3 Risk Factors Correlated With ATB in TAK Patients

In the univariate analysis (**Table 3**), hsCRP >8 mg/L (OR 3.131; 95% CI, 1.326–7.390; p=0.009) and positive T-SPOT.TB (OR 21.111; 95% CI, 5.018–88.822; p<0.001) were significantly correlated with the development of ATB in TAK patients. In the multivariate analysis, which included clinical variables with P value <0.10 in the univariate analysis, hsCRP >8 mg/L (OR 9.108; 95% CI, 1.096–75.711; p=0.041) and positive T-SPOT.TB (OR 68.669; 95% CI, 7.291–646.738; p<0.001) remained significantly correlated with the development of ATB.

**Table 3:** Univariate and multivariate analyses of the correlation between clinical variables and the development of ATB in TAK patients.

Variables	Odds ratio	Univariate analysis		Odds ratio	Multivariate analysis	
		95% CI	P value		95% CI	P value
Age (years)	1.004	0.970–1.040	0.823			
Male sex	1.714	0.672–4.372	0.259			
BMI (kg/m^2^)	0.965	0.871–1.068	0.488			
ESR >20 (mm/h)	1.710	0.743–3.937	0.207			
hsCRP >8 (mg/L)	3.131	1.326–7.390	0.009	9.108	1.096–75.711	0.041
Alb <40 (g/L)	2.000	0.843–4.747	0.116			
Positive T-SPOT.TB	21.111	5.018–88.822	<0.001	68.669	7.291–646.738	<0.001

### 3.4 Subsequent Surgical Interventions of TAK

The proportion of patients undergoing subsequent surgery for Takayasu arteritis was lower in patients with ATB (16.7 *vs.* 53.3%, p<0.001). Angiography was also performed more frequently in patients without ATB (6.7 *vs.* 27.8%, p=0.016) during follow-up.

## 4 Discussion

TAK has been noticed to be correlated with TB infection. We performed the first well-designed case-control study to evaluate the clinical features of TAK patients with ATB and to explore potential risk factors for ATB in TAK patients. The results indicated that ATB patients were more likely to develop several systemic symptoms. Elevated hsCRP level and positive T-SPOT.TB result were independent risk factors for ATB in TAK patients. In addition, subsequent surgical interventions for TAK patients might be hindered by ATB.

In the present study, participants of both groups were composed mostly by young females, consistent with the epidemiology of TAK (15, 16). TAK-related symptoms were usually determined by the corresponding involved site. Blood pressure could be elevated by the stenosis of renal artery and aorta, while peripheral vascular stenosis often causes dizziness, weak pulse, and blood pressure discrepancies between arms. As the NUMANO classification results did not differ between groups, patients with and without ATB shared similar TAK-related symptom profiles, suggesting limited contribution to the diagnosis of ATB by TAK-related symptoms. However, TAK patients with ATB are more prone to experience systemic symptoms including fever, fatigue, cough, expectorations, night sweating, and weight loss. Therefore, TB infection should be suspected and subsequent diagnostic tests should be performed clinically when the above symptoms occur.

Laboratory results were also potential biomarkers for the diagnosis of ATB in TAK patients. Compared with patients without ATB, patients with ATB had an elevated level of hsCRP, a declined level of albumin, and a higher positive rate of T-SPOT.TB test, reflecting the inflammatory and wasting nature of TB infection. By multivariate logistic regression analysis, hsCRP >8 mg/L and positive T-SPOT.TB result were further confirmed as independent risk factors for ATB in TAK patients. C reactive protein (CRP), an acute-phase reactant routinely tested on admission, reflects the innate immune response to infection. One study based on people living with HIV (PLWH) indicated that the hsCRP level could be utilized as a possible screening tool for ATB with a sensitivity of 90% and a specificity of 69% (17). Similar results were observed in other studies in which the combination of CRP and other biomarkers showed superior performance for TB identification (18, 19). However, a recent diagnostic accuracy study excluded the utility of CRP as a TB triage test due to its low specificity (20). Therefore, additional studies are still needed to assess the utility of CRP for TB screening, especially in TAK patients. Positive T-SPOT.TB was the other identified risk factor. T-SPOT.TB is an interferon-gamma release assay (IGRA) based on the MTB-specific T cell response with limited capability to differentiate LTBI and ATB (21). Yet, recent studies suggested that T cell immune response in ATB patients was significantly more vigorous, affirming the differential diagnostic value of T-SPOT.TB (22, 23).

Moreover, we also observed a significant decrease in the proportion of ATB patients undergoing subsequent surgery during follow-up, which was in accordance with the result of a previous large-sample retrospective study (11). The lower surgical rate indicated that ATB might hinder the subsequent surgical management of TAK patients. Surgical intervention was an integral component in the treatment of TAK, and therefore early recognition and management of patients with ATB could ensure adequate and timely integrated treatment for TAK patients.

The present study has several limitations. First, the retrospective nature and relatively small sample size might introduce a risk of bias to our results. Second, our hospital is a national referral center for complicated cases which guaranteed the accuracy of diagnosis and quality of treatment but could induce a potential selection bias at the same time. Third, the diagnosis of ATB in our study was acquired by comprehensive clinical evaluations from experienced experts of infectious diseases, and most of the patients were clinically diagnosed without microbiological evidence. However, several studies have suggested that microbiological examinations might be not that reliable as well (24, 25).

In conclusion, we reported the first case-control study to investigate potential risk factors for ATB in TAK patients. Our study revealed that the diagnosis of ATB should be considered when TAK patients experience systemic symptoms including fever, fatigue, cough, expectorations, night sweating, and weight loss. Moreover, hsCRP >8 mg/L and positive T-SPOT.TB result were identified as independent risk factors for ATB in TAK patients. Further prospective studies are warranted to verify our findings.

## Data Availability Statement

The raw data supporting the conclusions of this article will be made available by the authors, without undue reservation.

## Ethics Statement

This study complied with the Declaration of Helsinki and was approved by the Ethics Committee of PUMCH (ethics approval number: S-715). Informed consent was obtained from all patients enrolled.

## Author Contributions

Conceptualization: YC and YZ. Data curation: JZ, RJ, RZ, and JYZ. Formal analysis: RJ, JL, and XT. Funding acquisition: YC and YZ. Investigation: JZ. Methodology: RJ. Project administration: RZ. Resources: JYZ. Software: RJ. Supervision: YC and YZ. Validation: JZ. Visualization: JL and XT. Writing—original draft: JZ and RJ. Writing—review and editing: RZ, JYZ, JL, XT, YC, and YZ. All authors had full access to all the data in the study and had final responsibility for the decision to submit for publication. All authors contributed to the article and approved the submitted version.

## Funding

This work was supported by grant from the Major Research Program of Natural Science Foundation of China (grant number 51890894), grant from the Natural Science Foundation of China (grant numbers 81770481 and 82070492), and grant from Clinical Disease Diagnosis and Treatment Technology and Translational Research in Capital City (Z201100005520052).

## Conflict of Interest

The authors declare that the research was conducted in the absence of any commercial or financial relationships that could be construed as a potential conflict of interest.

## Publisher’s Note

All claims expressed in this article are solely those of the authors and do not necessarily represent those of their affiliated organizations, or those of the publisher, the editors and the reviewers. Any product that may be evaluated in this article, or claim that may be made by its manufacturer, is not guaranteed or endorsed by the publisher.
