# Supplementary material for: Clinical Features and Risk Factors for Active Tuberculosis in Takayasu Arteritis: A Single-Center Case-Control Study
Source: Front Immunol. 2021 Oct 29;12:749317. doi: 10.3389/fimmu.2021.749317 (PMC8586219; doi:10.3389/fimmu.2021.749317)
Supplement: Supplementary file 1 [file Table_1.docx]

**Table S1 Diagnosis criteria for ATB**

| Diagnosis | Criteria |
| --- | --- |
| Active tuberculosis (ATB) | a. Symptoms as follows: fever, fatigue, cough, night sweating, and weight loss, *etc.*  b. Laboratory results or radiological examinations supporting TB diagnosis.  c. Respond to anti-tuberculosis treatment.  d. Positive results of laboratory etiological detections (blood and sputum culture, fast acid staining, *etc*) and histological examinations (intestinal mucosal biopsy, *etc*).  Meeting a+d or a+b+c or both indicates ATB diagnosis. |
