# Supplementary material for: Clinical Features and Risk Factors for Active Tuberculosis in Takayasu Arteritis: A Single-Center Case-Control Study
Source: Front Immunol. 2021 Oct 29;12:749317. doi: 10.3389/fimmu.2021.749317 (PMC8586219; doi:10.3389/fimmu.2021.749317)
Supplement: Supplementary file 2 [file Table_2.docx]

**Table S2 Involved arteries of TAK patients with and without ATB**

| Involved arteries, n (%) | TAK with ATB (n = 30) | TAK without ATB (n = 90) | P value | |
| --- | --- | --- | --- | --- |
| Coronary artery | 0 (0.0%) | 2 (2.2%) | 1.000 | |
| Pulmonary artery | 6 (20.0%) | 12 (13.3%) | 0.385 | |
| Ascending aorta | 4 (13.3%) | 11 (12.2%) | 1.000 | |
| Aortic arch | 3 (10.0%) | 21 (23.3%) | 0.186 | |
| Brachiocephalic artery | 5 (16.7%) | 22 (24.4%) | 0.456 | |
| Common carotid artery | 9 (30.0%) | 42 (46.7%) | 0.137 | |
| Internal carotid artery | 2 (6.7%) | 12 (13.3%) | 0.513 | |
| Middle cerebral artery | 0 (0.0%) | 3 (3.3%) | 0.572 | |
| Subclavian artery | 13 (43.3%) | 49 (54.4%) | 0.302 | |
| Vertebral artery | 3 (10.0%) | 16 (17.8%) | 0.397 | |
| Axillary artery | 1 (3.3%) | 2 (2.2%) | 1.000 | |
| Thoracic aorta | 6 (20.0%) | 22 (24.4%) | 0.804 | |
| Abdominal aorta | 7 (23.3%) | 28 (31.1%) | 0.492 | |
| Celiac trunk | 2 (6.7%) | 11 (12.2%) | 0.514 | |
| Superior mesenteric artery | 4 (13.3%) | 15 (16.7%) | 0.779 | |
| Renal artery | 16 (53.3%) | 37 (41.1%) | 0.291 | |
| Common iliac artery | 0 (0.0%) | 4 (4.4%) | 0.571 | |
| Iliac artery | 1 (3.3%) | 3 (3.3%) | 1.000 | |
| Upper extremity artery | 1 (3.3%) | 2 (2.2%) | 1.000 |  |
| Lower extremity artery | 0 (0.0%) | 2 (2.2%) | 1.000 |  |
